# Supplementary material for: Semi-automated assembly of high-quality diploid human reference genomes
Source: Nature. 2022 Oct 19;611(7936):519–31. doi: 10.1038/s41586-022-05325-5 (PMC9668749; doi:10.1038/s41586-022-05325-5)
Supplement: Supplementary file 1 — This file contains Supplementary Notes 1-4, Supplementary Figures 1-10, Supplementary Table 14, and Supplementary references. [file 41586_2022_5325_MOESM1_ESM.pdf]

nature portfolio

https://doi.org/10.1038/s41586-022-05325-5

Supplementary information

Semi-automated assembly of high-quality diploid human reference genomes

In the format provided by the authors and unedited

Semi-automated assembly of high-quality diploid human reference genomes

Jarvis, Formenti et al

Supplementary Notes

Supplementary Note 1: Estimation of diversity representation
Based on theoretical estimates from SNPs in the 1000G project, and using Kruglyak et al 2000 formula[1], to detect 100% of minor alleles greater than 2% in the human world population or ~97% of those above 1%, one would need to generate complete sequence for ~350 diploid genomes (700 haplotypes) from diverse populations, the number thus far funded. To obtain 99% of those above 1%, one would need ~450 genomes (900 haplotypes). Minor allele frequency is the frequency at which the second most common allele occurs in a population. This calculation will differ for structural variants, estimated at 50 genomes for alleles above 5%. These values will need to be revised in an iterative fashion, as more relatively complete and error-free genomes are generated. For example, as validated in this study, haplotype diversity is uneven across the genome, e.g. where centromeres have much greater diversity than most other genic parts of the genome. Also the technology used will make a difference. These values were determined based mostly on short-read only assemblies, which do not assemble centromeres well, and also not GC-rich or repeat regions well.

Supplementary Note 2: Additional reads with adaptors that needed to be removed.
After the standard method of removing adaptors in the sequence reads, we screened the presence of any remaining adaptors in the HG002 Pacbio libraries. We found that 0.3-0.4% of reads (~2,000) still contained at least 1 adapter per SMRT cell movie, and that adds up to a 1.7% frequency overall (all contaminated reads/all reads). Such adaptor contaminated reads were greatly reduced with chemistry 3.0 relative to 2.0 and pre2.0. The PacBio 45 bp blunt adapter was the sequence that was not properly clip in the sequence processing:

> gnl|uv|NGB00972.1:1-45 Pacific Biosciences Blunt Adapter
ATCTCTCTCTTTTCCTCCTCCTCCGTTGTTGTTGTTGAGAGAGAT

These were due to reads with adapter 2-dimers (more prevalent in chem pre2.0 than in chem2.0). The location of the adapters were mostly (90%) at the beginning of the reads (first 1/3rd of the read length), and 87% within the first 100bp; 0.1% in the middle of the read (2/3 of the read); and the remaining 9.5% at the end of the read (last third of the read), and 9.3 % within the last 100bp. To identify reads with reads of all these categories, we used HiFiAdapterFilt (https://github.com/sheinasim/HiFiAdapterFilt). Some reads were entirely made of adaptors, which presumably occurs by self ligation in the library preparation. We removed all such reads with adaptors for the final HG002 assembly, even if they had human sequence attached, because using cut versions of these reads led to lower assembly metrics relative to without them. Later we switched to CutAdapt (https://github.com/marcelm/cutadapt)[3], as the results had about 90% overlap with HiFiAdapterFilt, but Cutadapt was about 2 times faster and slightly more sensitive (5-10%).

Supplementary Note 3. Gene set used for determining gene presence or absence in the reference assemblies. The assessment of gene content was done by aligning known RefSeq transcripts (with accession prefixes NM_ or NR_) for human genes to all assemblies. This set of transcripts is actively maintained by the NCBI RefSeq group based on experimental data (genome sequence, and transcript and protein products). Over 97% of RefSeq known transcripts have been curated and are in status “REVIEWED” or “VALIDATED” (2 of the 7 most evidence based categories; https://www.ncbi.nlm.nih.gov/books/NBK21091/table/ch18.T.refseq_status_codes/?report=objectonly).

The absent genes from one of the four reference haplotypes (GRCh38, CHM13, HG002 maternal and HG002 paternal) are enriched (38%) for status “PROVISIONAL” due to microRNAs, which were originally predicted by miRbase but have yet to be validated. Excluding miRNAs, nearly 90% of the transcripts not found in one of the four reference assemblies have been curated and are therefore unlikely to be artefacts.

Supplementary Note 4. In a separate sample HG06807 (an African American female), we compared genome contigs generated using hifiasm (v0.15.5) on HiFi reads (~30x coverage) from LCL versus blood of the same individual. We called variants between the two assemblies with Dipcall, using minimap2 alignments to GRCh38. The intersection of Dipcall confident BED files were used to include only the variants from the well-assembled and well-aligned blocks. VCF comparisons were done w/ hap.py
hap.py ${BLOOD_VCF} ${CELL_VCF} -f ${CONFIDENT_BED} -r ${REF} -o ${OUTPUT_PREFIX} --pass-only --no-roc --no-json --engine=vcfeval --engine-vcfeval-template=${REF_SDF}. The variants that couldn't be compared by vcfeval (because of complexity) are excluded. The intersected DipCall confident blocks cover ~2.79 Gb (90%) of GRCh38 (3.09 Gb); the projection of the confident blocks also covers ~90% of each of the HG002 maternal and paternal assemblies.

We found that the LCL-based and blood-based SNVs are highly similar (99.8%; **Supplementary Table 14**). That is, 99.8% of the LCL SNVs were also present in blood and 99.8% of the blood SNVs were also present in the LCL. The ~10,000-12,000 SNVs present only in the LCL or blood could be due to real mosaicism substitutions, or a combination of mosaicism and consensus sequence read errors in hifiasm contigs of HiFi reads (QV59 would be mean 3000-4000 errors in a 3Gb genome). We also searched for common and different variants that intersected with the ClinVar database and we could not find any pathogenic variants.

To search for potential SV differences in the blood versus the LCL, we aligned the LCL contig assembly to the blood contig assembly (as reference) per haplotype separately with minimap2-v2.24. For each haplotype we then ran SVIM-asm, called all potential SVs per haplotype, and filtered out putative SVs that included gaps in the assembly of any of the haplotypes, indicating possible assembly errors. From this analysis, we found three small inversions (two on the same chromosome) ranging from ~1.6, 4.0, to 10 Kb in the maternal haplotype of the LCL relative to the original blood source (**Supplementary Fig. 9**), and not in the paternal haplotype; thus, these inversions without gaps appear not to be due to assembly errors. The combined SNV and SV findings suggest that low passage LCLs may not be identical to the original blood source. However, they have not introduced major changes in the genome relative to their starting blood source.

Type	Blood	LCL	Only Blood	Only LCL	Common	% Common
SNVs	4,724,714	4,723,159	12,307	10,752	4,712,407	99.8%, 99.8%

Supplementary Table 14. SNVs relative to GRCh38 in blood versus LCL genome of the same individual. The two % common values are the percentage of shared variants in the blood and LCL, respectively, relative to GRCh38.

Supplementary Figures

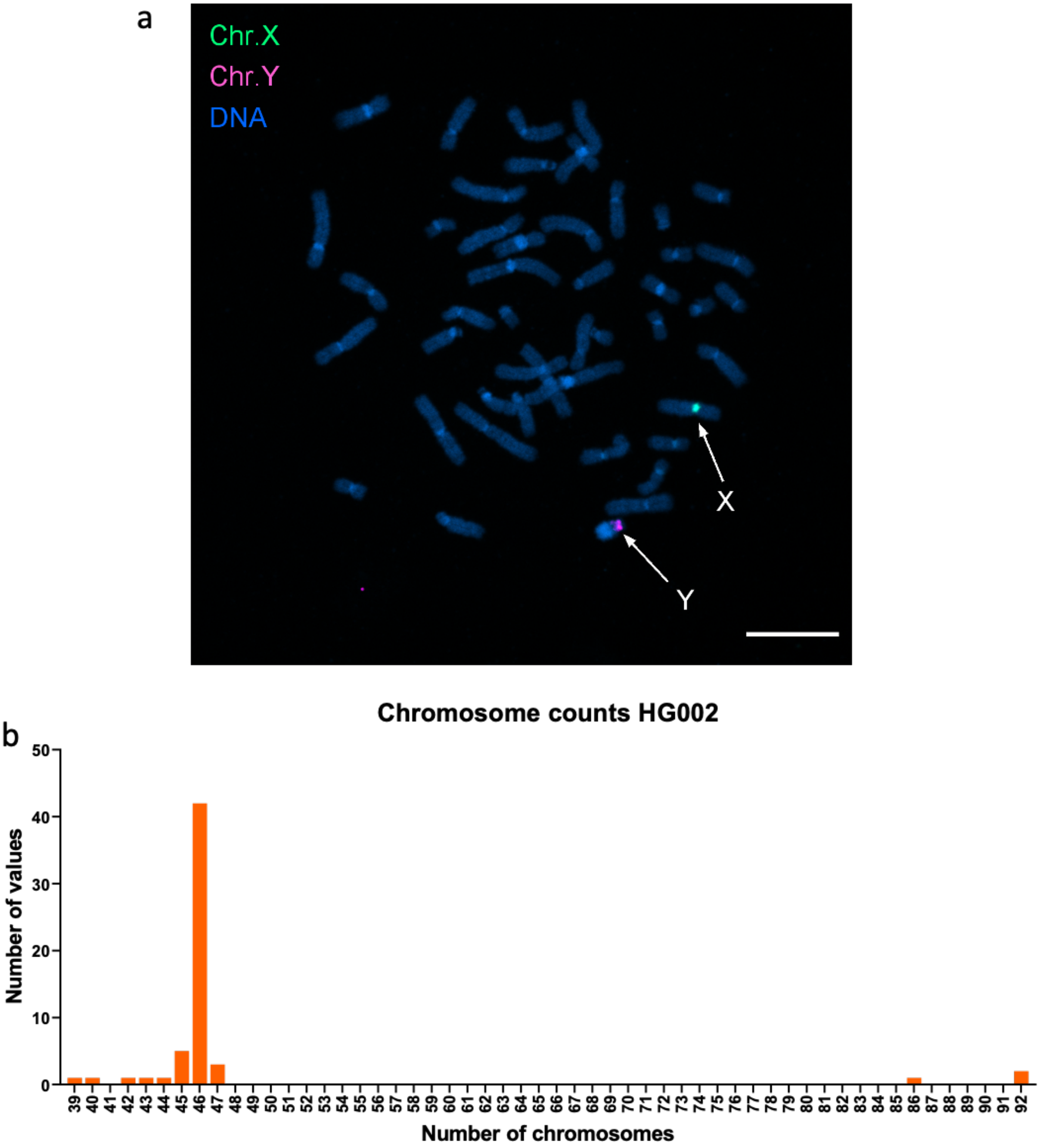

Supplementary Fig. 1 | Ploidy analysis of HG002 LCL GM24385. a, Representative chromosome spread of passage 13 LCLs demonstrating 46, XY karyotype. Chromosomes were labeled by FISH with X centromeric probe for DXZ1 satellite (green) and a custom Y chromosome probe from in-house single-sorted chromosomes (pink)[2]; the DNA was counter-stained with DAPI. Scale bar, 10um **b**, Ploidy distribution of 58 randomly selected chromosome spreads from HG002 LCLs showed an expected modal number of 46 chromosomes. Spreads missing multiple chromosomes may be specimen preparation artifacts. Overall, most cells maintain a stable diploid karyotype.

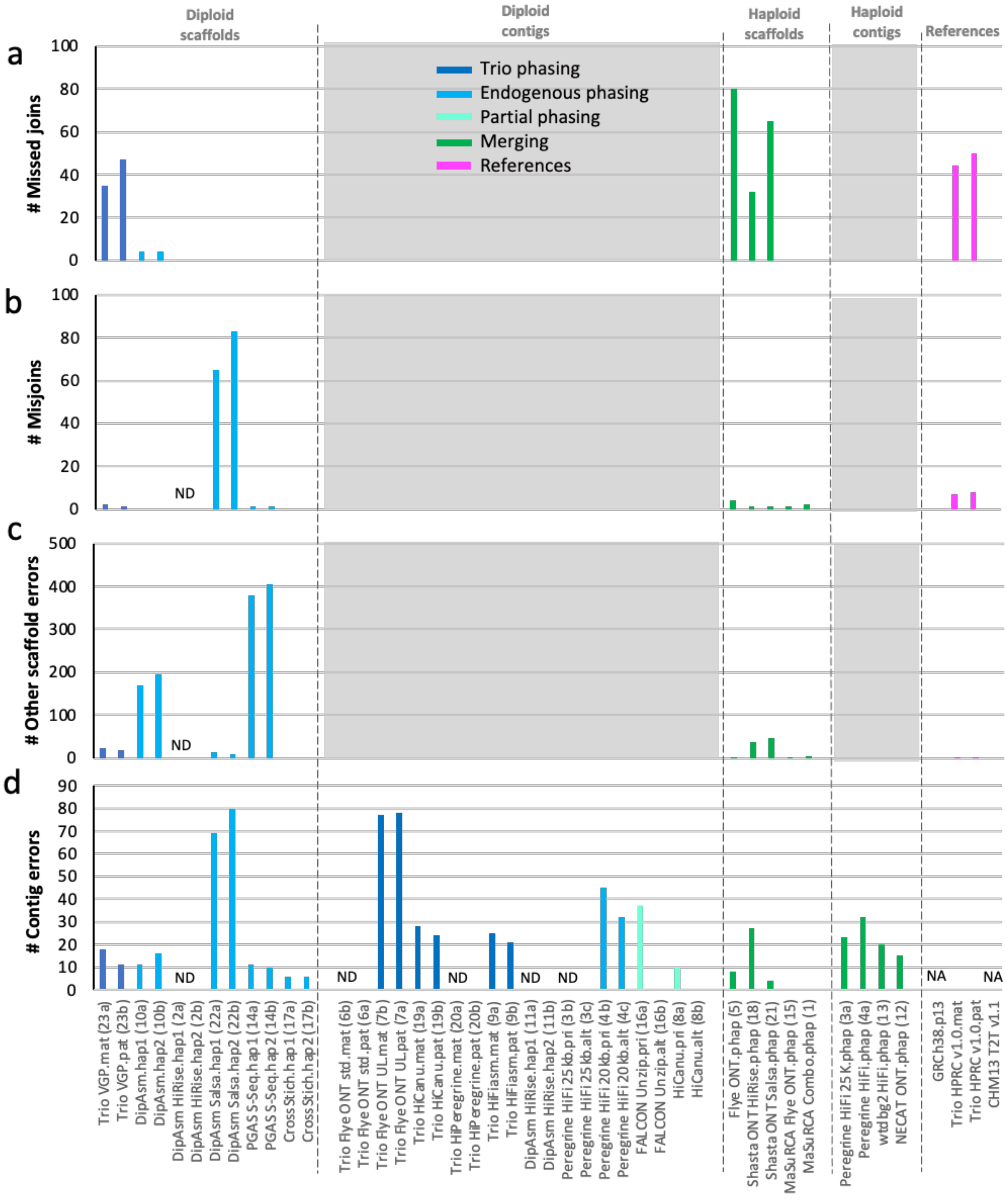

Supplementary Fig. 2 | Curation structural analyses. a, Missed contig joins that should have been brought together in the same scaffold. **b**, Misjoins of contigs that should not have been brought together. **c**, other scaffold errors, including false inversions and false duplications. **d**, Contig errors, including chimeric contigs. ND = not done, as it would be redundant with another assembly approach. NA = Not applicable, as these references were curated by a different process, with nevertheless many errors corrected.

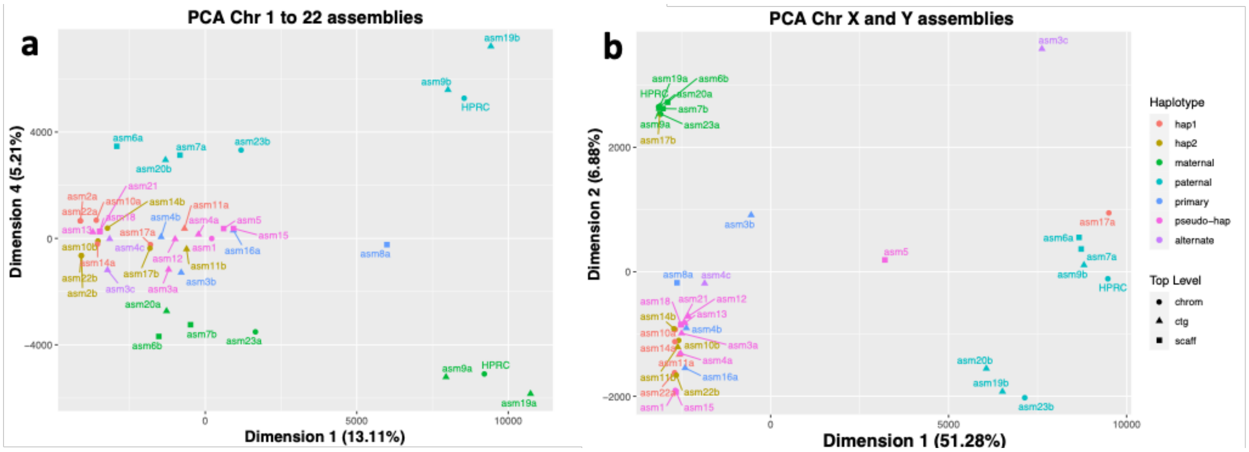

.

Supplementary Fig. 3 | a and **b**, PCA on the multidimensional Euclidean distances amongst assemblies, for autosomes 1-22 and sex chromosomes X and Y, respectively from Figure 2c,d, but with simpler assembly labels. The labels are simplified in order to better see the location of the values in the graphs. The haplotypes of trio-based assemblies (green for maternal; turquoise for paternal) are the most distinguished from all other assemblies and from each other.

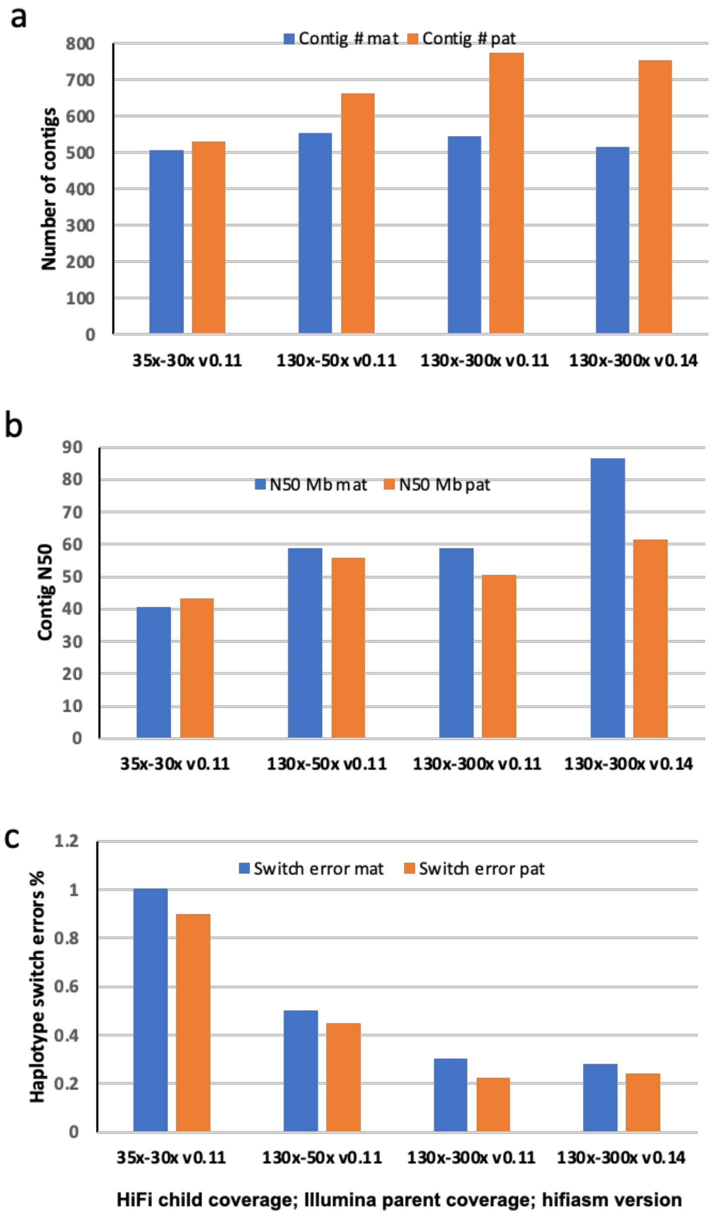

Supplementary Fig. 4. | **Titration of read coverage and hifiasm version on three assembly metrics. a**, Number of contigs for each haplotype. **b**, Contig N50 for each haplotype. **c**, Haplotype switch errors for each haplotype. Two HiFi coverage levels were tested: 35x and the near full 130x data set with different library insert sizes. Three parental Illumina coverage levels were tested: 30x; 50x; and 300x. Two trio hifiasm versions were tested: v0.11 and 0.14. The increase in the number of contigs with increasing child and parental coverage could be due to more complete contigs with diverse centromere sequences, human EBV virus, and mitochondrial genomes. Hifiasm v0.14 increased contig N50. Increased child or parental sequence coverage decreased haplotype switch errors. The 130x-300x v0.14 assembly was the contig input for the final HPRC-HG002 assembly of this study.

b, Sequences of maternal gaps filled, 10 of them

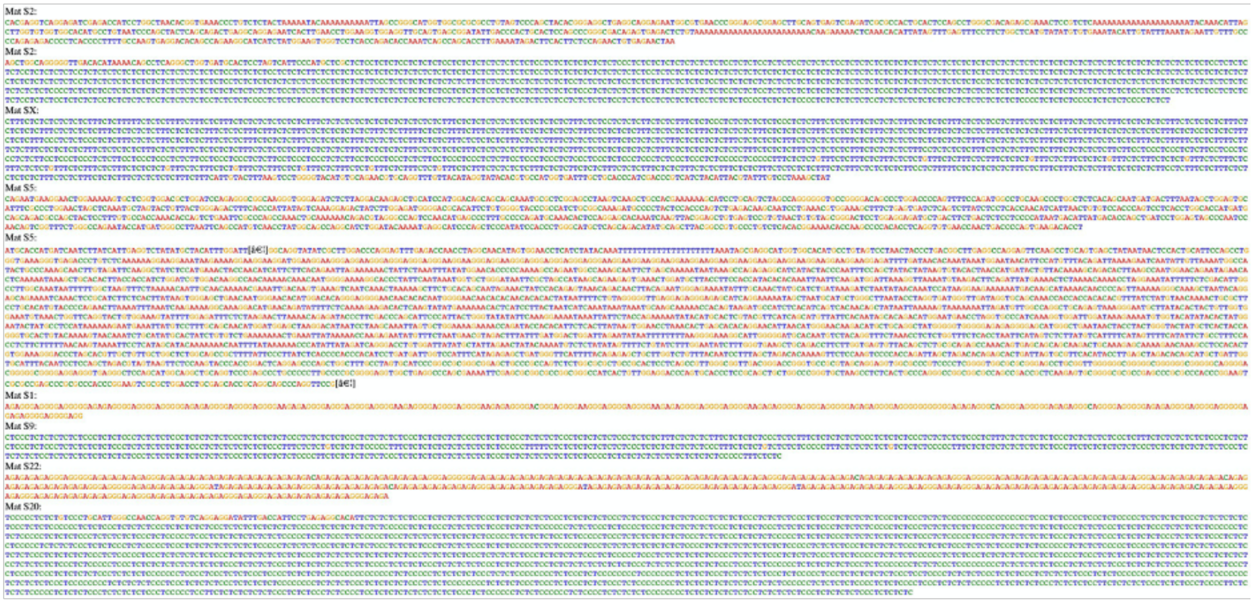

a, Sequences of paternal gaps filled, 5 of them

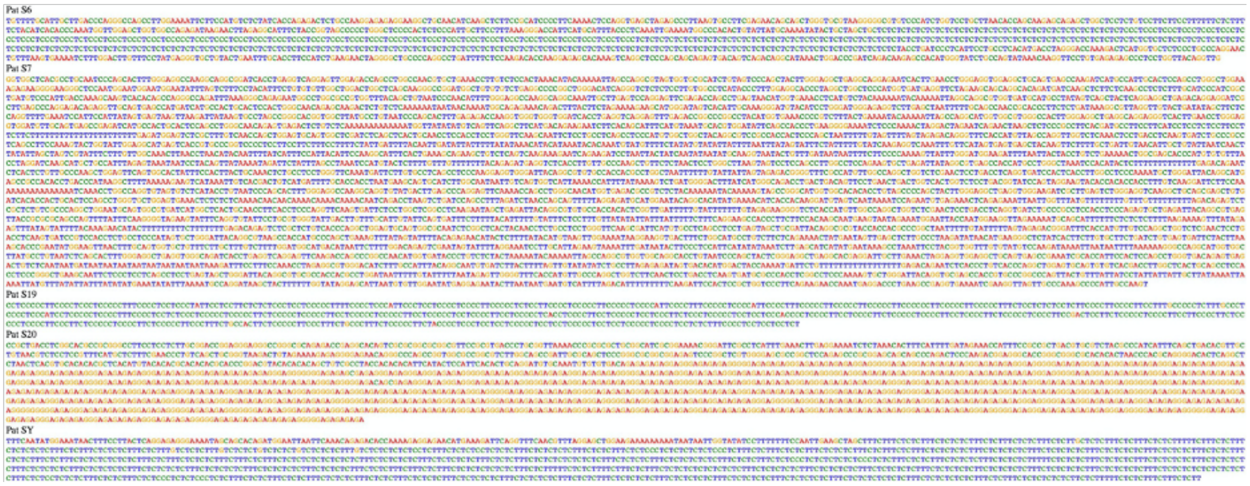

Supplementary Fig. 5 | Gap filled sequences. **a**, Sequences filled in the maternal scaffolds. **b**, Sequences filled in the paternal scaffolds. Only the sequences used in the gap space are shown. These gaps between Pacbio HiFi-based contigs in scaffolds were filled with ONT-based contigs. Orange = G; Red = A; Green = C; Blue = T. Notice the GA or TC repeats in most of the filled gap sequence.

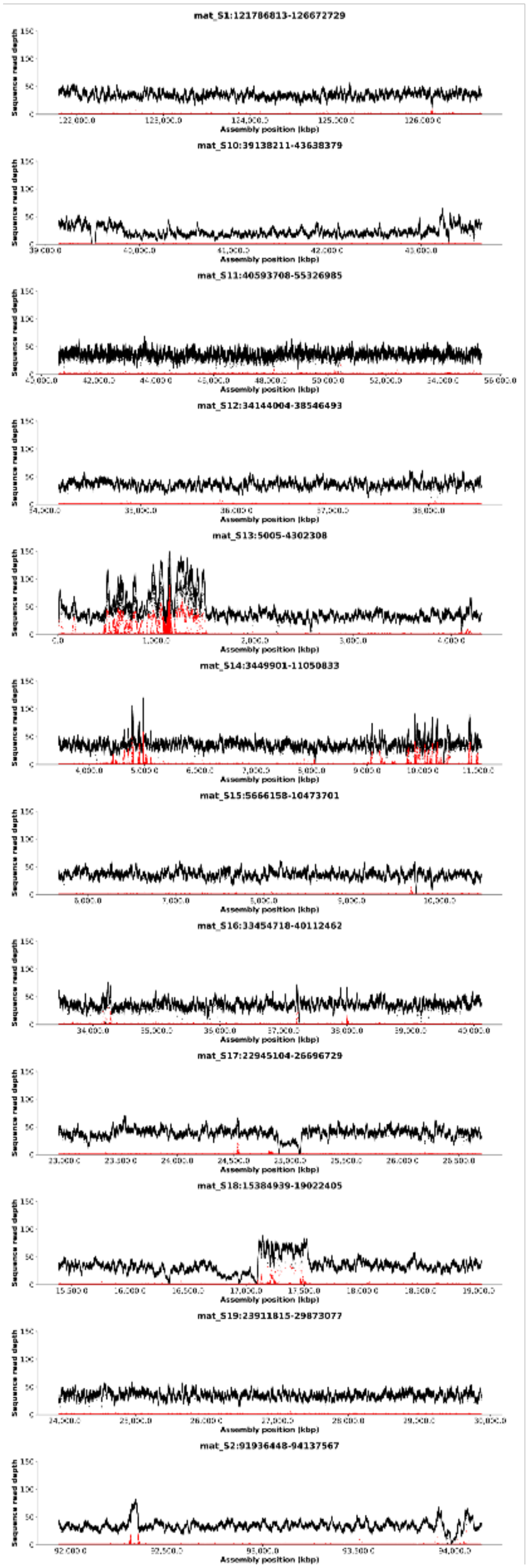
mat_S1:121786813-126672729
mat_S10:39138211-43638379
mat_S11:40593708-55326985
mat_S12:34144004-38546493
mat_S13:5005-4302308
mat_S14:3449901-11050833
mat_S15:5666158-10473701
mat_S16:33454718-40112462
mat_S17:22945104-26696729
mat_S18:15384939-19022405
mat_S19:23911815-29873077
mat_S2:91936448-94137567
Sequence read depth
Assembly position (kbp)

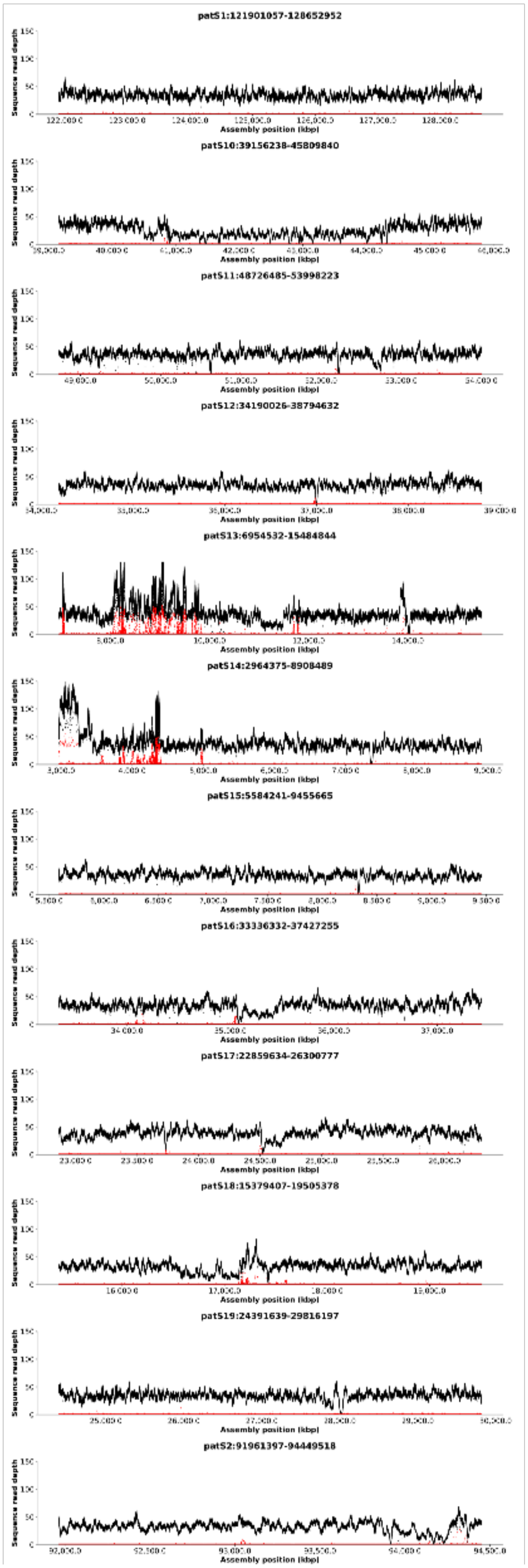
patS1:121901057-128652952
patS10:39156238-45809840
patS11:48726485-53998223
patS12:34190026-38794632
patS13:6954532-15484844
patS14:2964375-8908489
patS15:5584241-9455665
patS16:33336332-37427255
patS17:22859634-26300777
patS18:15379407-19505378
patS19:24391639-29816197
patS2:91961397-94449518
Sequence read depth
Assembly position (kbp)

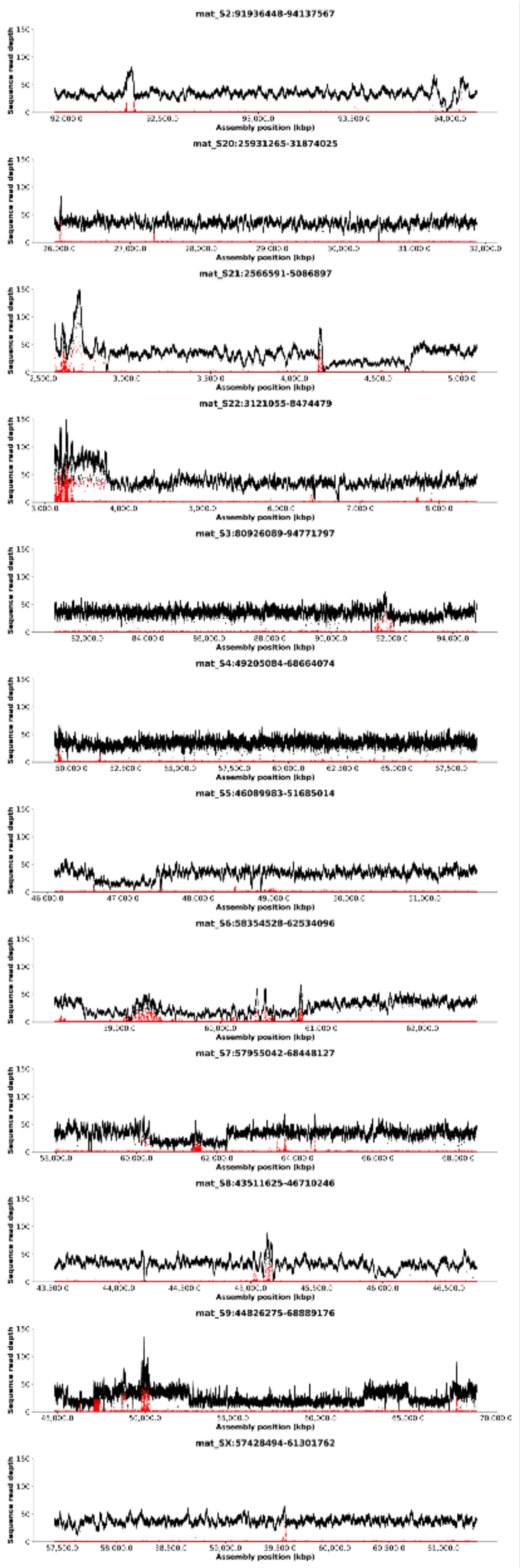
mat_S2:91936448-94137567
mat_S20:25931265-31874025
mat_S21:2566591-5086897
mat_S22:3121055-8474479
mat_S3:80926089-94771797
mat_S4:49205084-68664074
mat_S5:46089983-51685014
mat_S6:58354528-62534096
mat_S7:57955042-68448127
mat_S8:43511625-46710246
mat_S9:44826275-68889176
mat_SX:57428494-61301762
Sequence read depth
Assembly position (kbp)

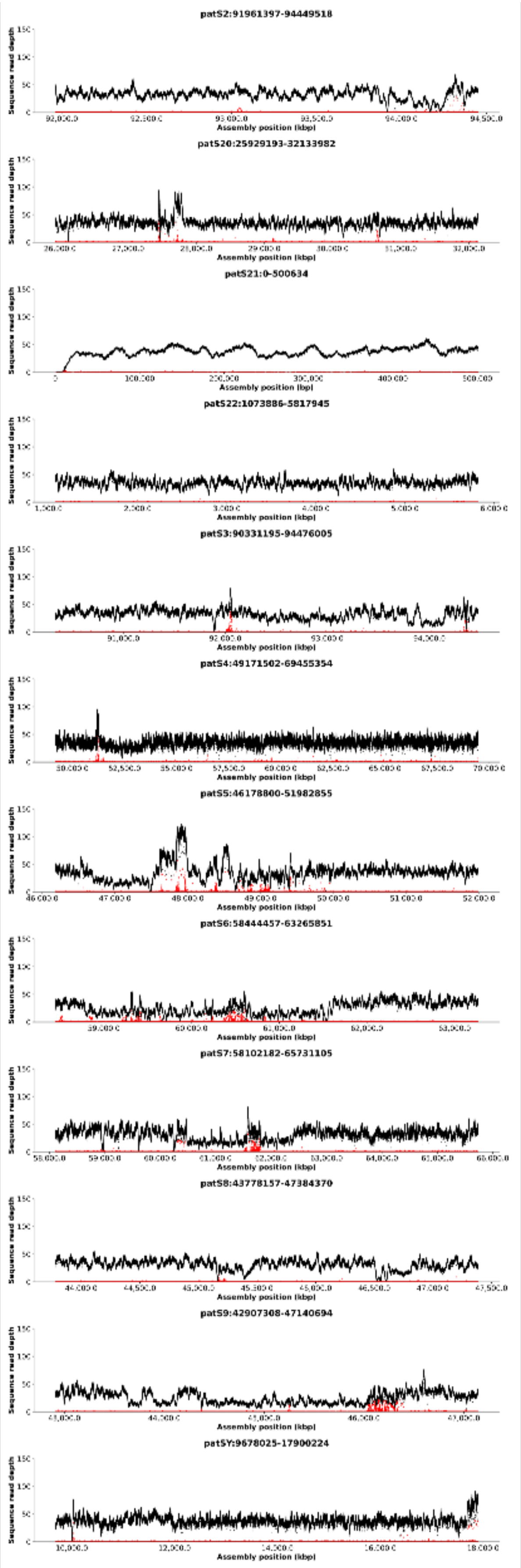
patS2:91961397-94449518
patS20:25929193-32133982
patS21:0-500634
patS22:1073886-5817945
patS3:90331195-94476005
patS4:49171502-69455354
patS5:46178800-51982855
patS6:58444457-63265851
patS7:58102182-65731105
patS8:43778157-47384370
patS9:42907308-47140694
patSY:9678025-17900224
Sequence read depth
Assembly position (kbp)
Assembly position (bp)

Supplementary Fig. 6. Assessing centromere collapses. Shown are HiFi coverage plots across the alpha satellite array regions of the centromeres in the HRPC-HG002 v1.0 maternal (left) and paternal (right) assemblies. A collapsed repetitive region will have coverage pile up that is two or more times higher than the mean coverage of the surrounding genomic region. These regions can also include alleles from the other haplotype (red), which could result in switch errors. Regions of decreased coverage or dropout in coverage could also have switch errors associated with them or are more difficult to sequence through.

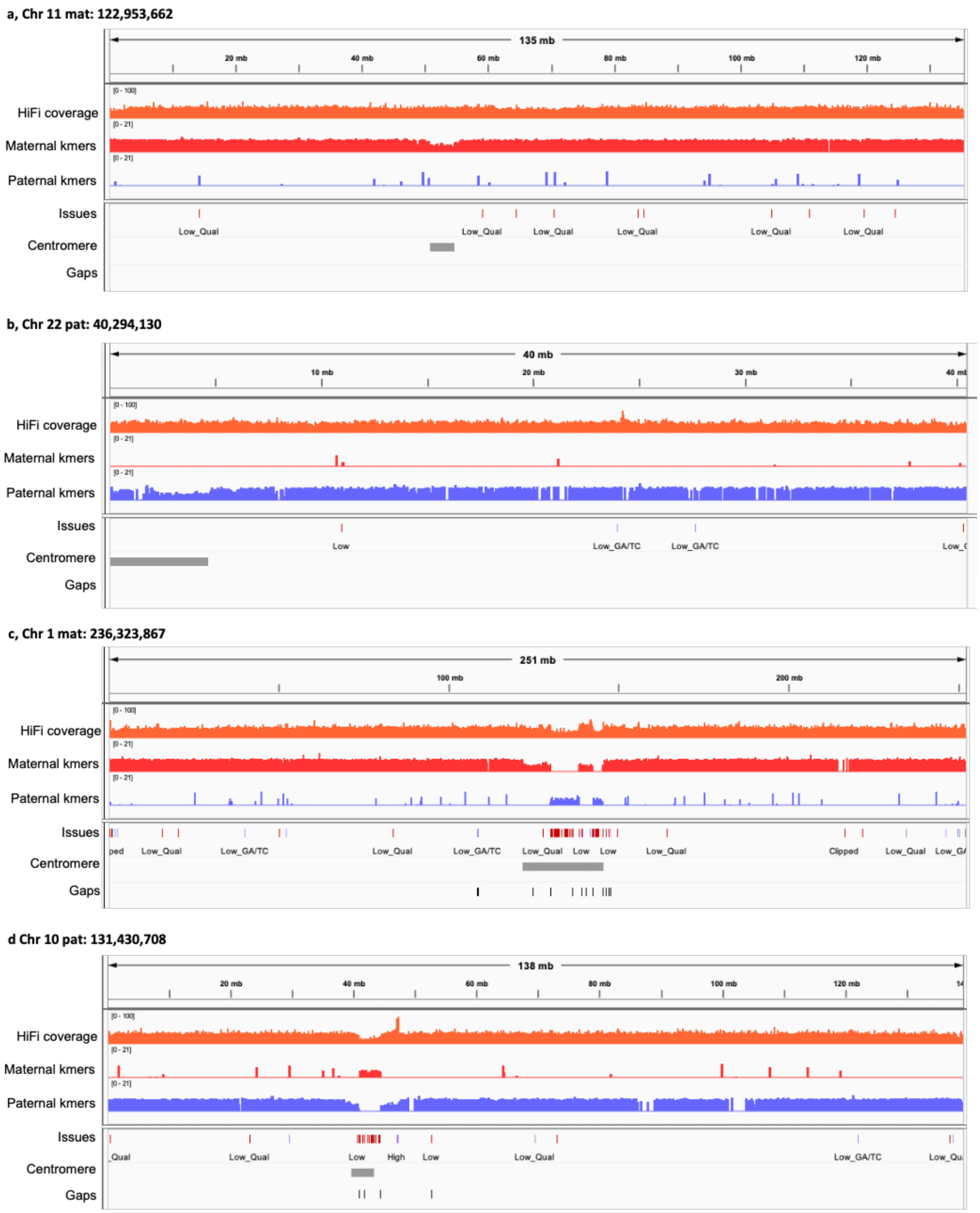

Supplementary Fig. 7 | Assessing centromere switch errors. a-b, Examples of assembled HPRC-HG002 v1.0 centromere regions with no gaps or haplotype switch errors. **c-d**, Examples with gaps and haplotype switch errors. Top row, HiFi coverage across the chromosome segment; below, maternal (red) and paternal (blue) *k-mer* profiles, where haplotype switches show up as swapping in k-mer coverage. Issues, indicate low quality sequence or other potential problems. Grey bar, centromere. Gaps, are black tick marks.

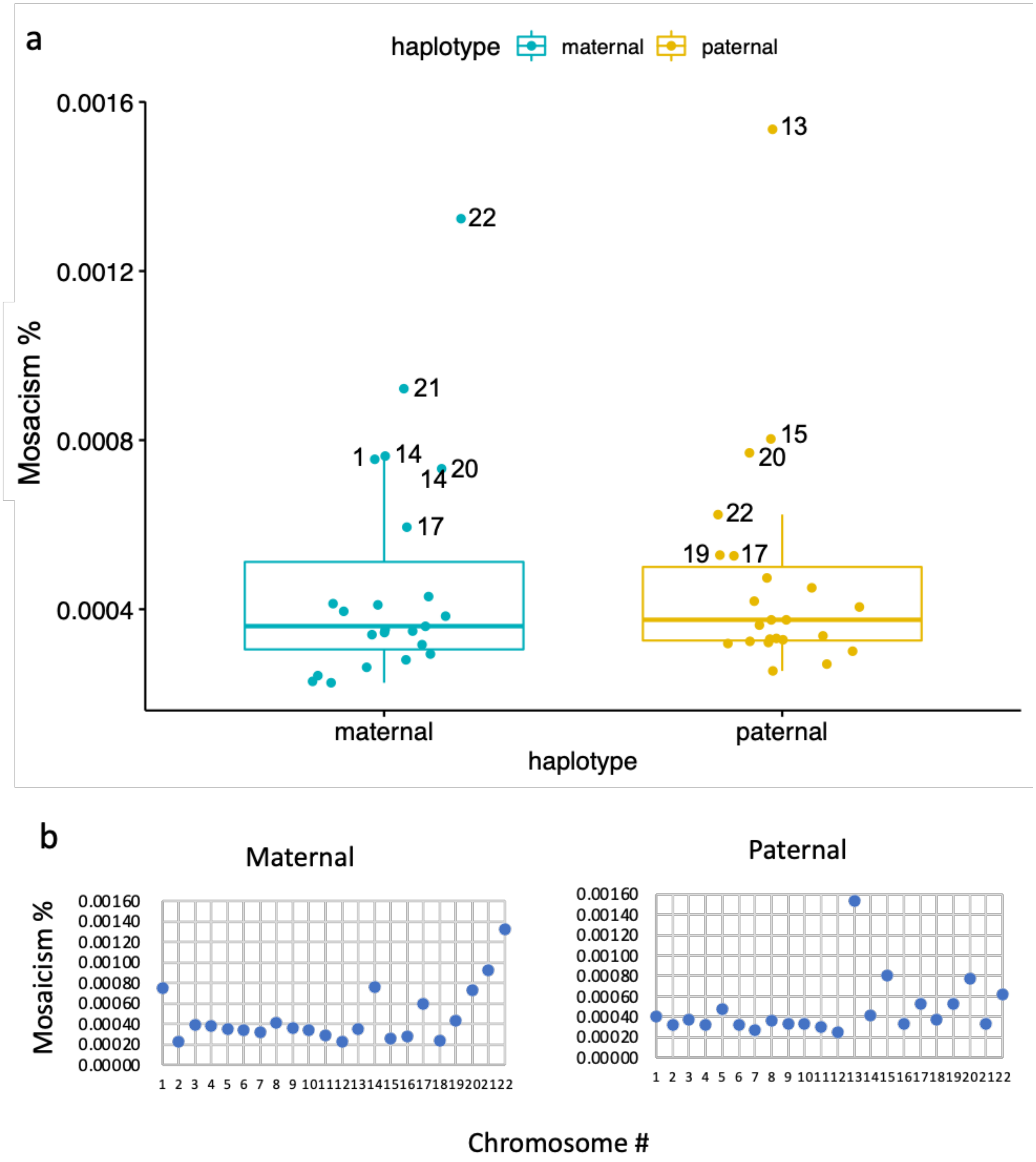

Supplementary Fig. 8. Mosaicism in HG002 haplotypes. a, Graphed are the rate of minor alleles in the raw reads found for each haplotype. Each dot represents the value for each autosome. Chromosomes with mosaicism higher than the 95% confidence interval are numbered. **b**, Mosaicism relative to chromosome size, with chromosomes ordered from largest to smallest. Note that a higher prevalence of mosaicism among the smaller chromosomes.

a, Maternal Inversion 1

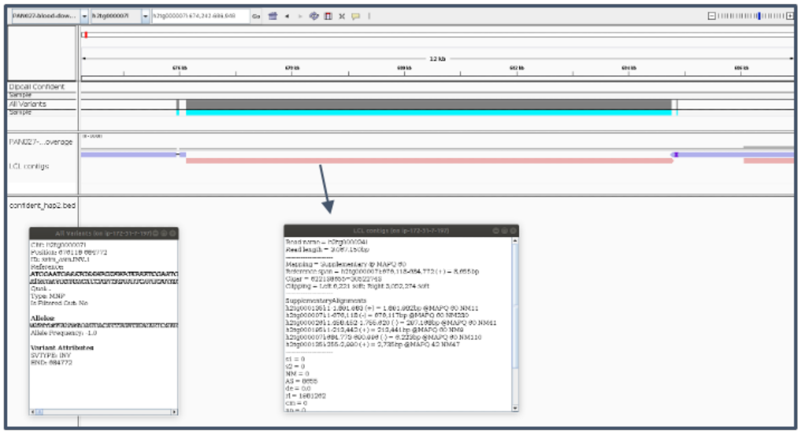

b, Maternal Inversion 2

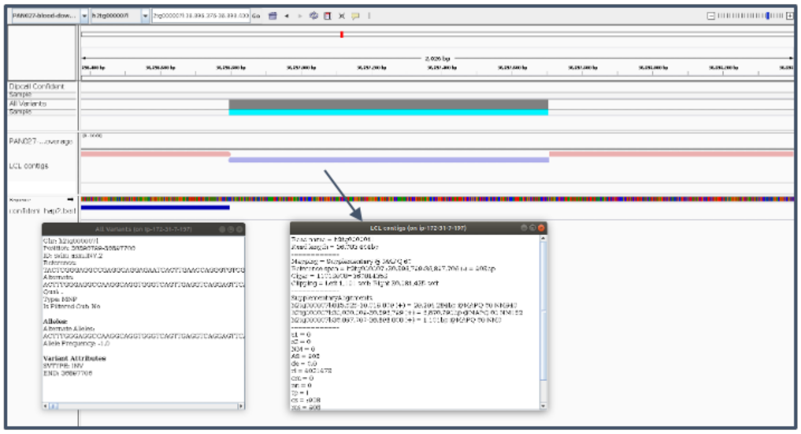

c, Maternal Inversion 3

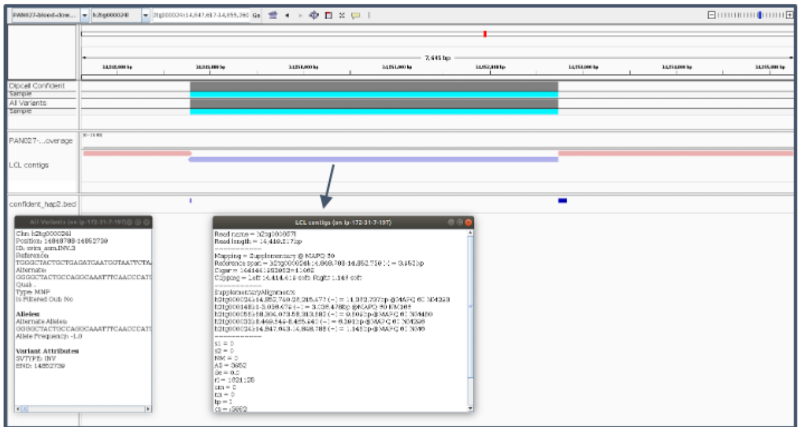

Supplementary Fig. 9. Several structural variant inversion differences in blood versus LCL of a human sample. **a,** IVG plot of ~10 kb inversion in a contig of the maternal haplotype of the LCL sample aligned to blood sample contigs. **b,** Plot of ~1.6 kb inversion in another region of the same contig as in (a). **c,** Plot of ~4 kb inversion in a different contig of the maternal LCL haplotype.

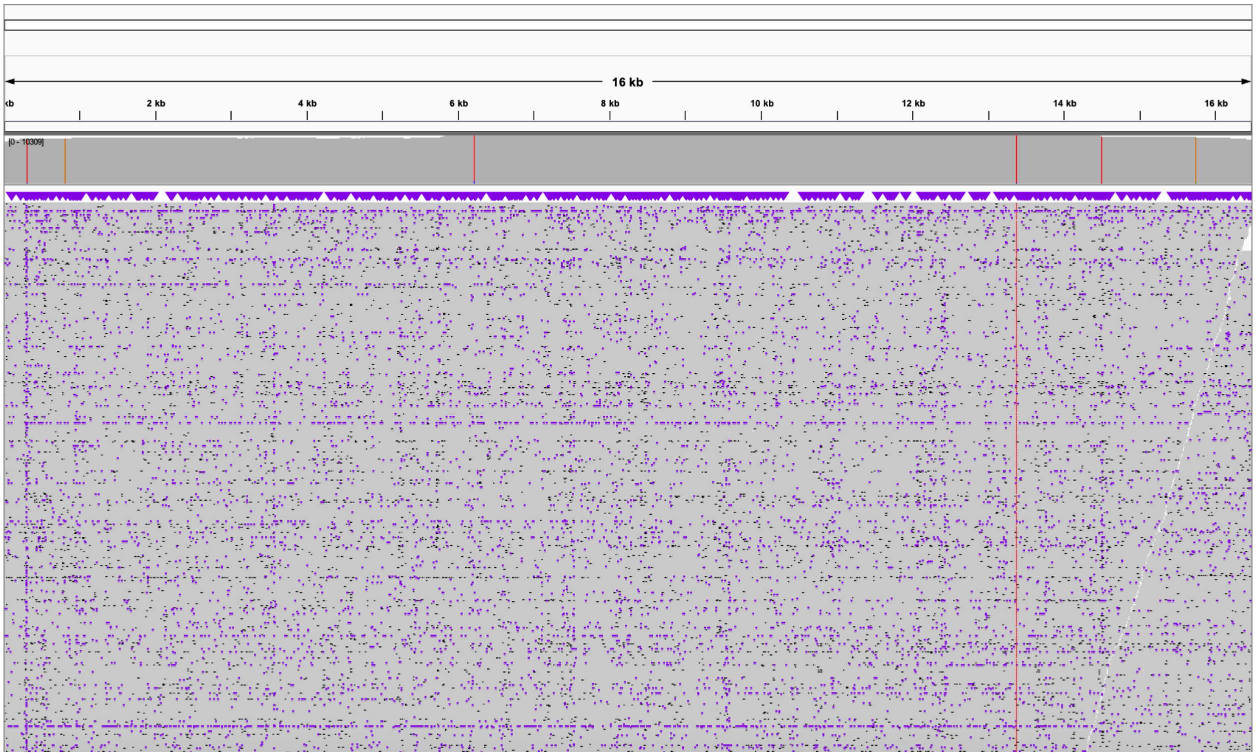

Supplementary Fig. 10. Mitochondrial genome heteroplasmy in HG002 cell line. Shown are raw HiFi reads mapped back to the reference HG002 mitochondrial genome assembly. Red vertical lines indicate SNPs at a frequency of >1% The one red line prominent in the raw reads indicates that the minor allele was in the reference. This heteroplasmy could have been presented in the original blood cell plasma isolated from HG002 and/or in the subsequent cell line.

References

1. Kruglyak, S., Durrett, R., Schug, M. D. & Aquadro, C. F. Distribution and abundance of microsatellites in the yeast genome can Be explained by a balance between slippage events and point mutations. *Mol. Biol. Evol.* **17**, 1210–1219 (2000).
2. Potapova, T. A. *et al.* Karyotyping human and mouse cells using probes from single-sorted chromosomes and open source software. *BioTechniques* **59**, 335–336, 338, 340-342 passim (2015).
3. Martin, M. Cutadapt removes adapter sequences from high-throughput sequencing reads. *EMBnet.journal* **17**, 10 (2011).
