# Supplementary material for: Semi-automated assembly of high-quality diploid human reference genomes
Source: Nature. 2022 Oct 19;611(7936):519–31. doi: 10.1038/s41586-022-05325-5 (PMC9668749; doi:10.1038/s41586-022-05325-5)
Supplement: Supplementary file 2 — Reporting Summary [file 41586_2022_5325_MOESM2_ESM.pdf]

nature portfolio

Corresponding author(s): Erich Jarvis, Giulio Formenti, Kerstin Howe, Karen Miga

Last updated by author(s): Jul 2, 2022

Reporting Summary

Nature Portfolio wishes to improve the reproducibility of the work that we publish. This form provides structure for consistency and transparency in reporting. For further information on Nature Portfolio policies, see our Editorial Policies and the Editorial Policy Checklist.

Statistics

For all statistical analyses, confirm that the following items are present in the figure legend, table legend, main text, or Methods section.

n/a	Confirmed	
☐	☒	The exact sample size (*n*) for each experimental group/condition, given as a discrete number and unit of measurement
☐	☒	A statement on whether measurements were taken from distinct samples or whether the same sample was measured repeatedly
☐	☒	The statistical test(s) used AND whether they are one- or two-sided<br>*Only common tests should be described solely by name; describe more complex techniques in the Methods section.*
☒	☐	A description of all covariates tested
☒	☐	A description of any assumptions or corrections, such as tests of normality and adjustment for multiple comparisons
☐	☒	A full description of the statistical parameters including central tendency (e.g. means) or other basic estimates (e.g. regression coefficient) AND variation (e.g. standard deviation) or associated estimates of uncertainty (e.g. confidence intervals)
☐	☒	For null hypothesis testing, the test statistic (e.g. *F*, *t*, *r*) with confidence intervals, effect sizes, degrees of freedom and *P* value noted<br>*Give P values as exact values whenever suitable.*
☒	☐	For Bayesian analysis, information on the choice of priors and Markov chain Monte Carlo settings
☒	☐	For hierarchical and complex designs, identification of the appropriate level for tests and full reporting of outcomes
☒	☐	Estimates of effect sizes (e.g. Cohen's *d*, Pearson's *r*), indicating how they were calculated

Our web collection on statistics for biologists contains articles on many of the points above.

Software and code

Policy information about availability of computer code

Data collection	All code used to collect data are cited in github links and citations of the manuscript
Data analysis	All code used to analyzes the data are cited in github links and citations of the manuscript

For manuscripts utilizing custom algorithms or software that are central to the research but not yet described in published literature, software must be made available to editors and reviewers. We strongly encourage code deposition in a community repository (e.g. GitHub). See the Nature Portfolio guidelines for submitting code & software for further information.

Data

Policy information about availability of data

All manuscripts must include a data availability statement. This statement should provide the following information, where applicable:

- Accession codes, unique identifiers, or web links for publicly available datasets
- A description of any restrictions on data availability
- For clinical datasets or third party data, please ensure that the statement adheres to our policy

All raw sequence data used in this study is available at the following HPRC Github: https://github.com/human-pangenomics/HG002_Data_Freeze_v1.0. The final HPRC-HG002 curated assemblies are available in NCBI under the BioProject IDs PRJNA794175 and PRJNA794172, with accession numbers GCA_021951015.1 and GCA_021950905.1, for the maternal and paternal haplotypes respectively.

March 2021

Human research participants

Policy information about studies involving human research participants and Sex and Gender in Research.

Reporting on sex and gender	The biological sex (male and female) of the samples used have been noted, as our goal was to include genome sequencing and assembly of both X and Y sex chromosomes.
Population characteristics	The population that the individuals are from (Ashkenazi, African American) have been noted, and approved in the original informed consents.
Recruitment	Participants were recruited in separate studies from this study
Ethics oversight	Personal Genome Project (HG002) and Washington University (HG06807)

Note that full information on the approval of the study protocol must also be provided in the manuscript.

Field-specific reporting

Please select the one below that is the best fit for your research. If you are not sure, read the appropriate sections before making your selection.

☒ Life sciences ☐ Behavioural & social sciences ☐ Ecological, evolutionary & environmental sciences

For a reference copy of the document with all sections, see nature.com/documents/nr-reporting-summary-flat.pdf

Life sciences study design

All studies must disclose on these points even when the disclosure is negative.

Sample size	No sample size estimate was necessary. This is a high-quality reference genome sequencing project, focused on one individual as representative of the species. The variation that exist within humans is not expected to dramatically change the outcome of the findings.
Data exclusions	No data were excluded from the analyses
Replication	Most algorithms applied in this study were repeated two or more times on the same sample
Randomization	Randomization is not applicable to this genome assembly study, as there is a sample size of one individual.
Blinding	Blinding is not applicable, as there are not any quantifications that are subjective.

Reporting for specific materials, systems and methods

We require information from authors about some types of materials, experimental systems and methods used in many studies. Here, indicate whether each material, system or method listed is relevant to your study. If you are not sure if a list item applies to your research, read the appropriate section before selecting a response.

Materials & experimental systems

n/a	Involved in the study	
☒	☐	Antibodies
☐	☒	Eukaryotic cell lines
☒	☐	Palaeontology and archaeology
☒	☐	Animals and other organisms
☒	☐	Clinical data
☒	☐	Dual use research of concern

Methods

n/a	Involved in the study	
☒	☐	ChIP-seq
☒	☐	Flow cytometry
☒	☐	MRI-based neuroimaging

Eukaryotic cell lines

Policy information about cell lines and Sex and Gender in Research

Cell line source(s)	GM24385 (RRID:CVCL_1C78) EBV-immortalized lymphoblastoid cell line (LCL) of HG002
Authentication	Genome sequence authentication

Mycoplasma contamination	None detected in the genome data
Commonly misidentified lines<br>(See ICLAC register)	*Name any commonly misidentified cell lines used in the study and provide a rationale for their use.*
